# Correspondence on “piperine as a potential molecule for disease prevention and management

**DOI:** 10.1186/s43088-022-00226-y

**Published:** 2022-03-28

**Authors:** Rujittika Mungmunpuntipantip, Viroj Wiwanitkit

**Affiliations:** 1 Bangkok, Thailand; 2 Dr DY Patil University, Pune, India

Dear Editor,

 We would like to share ideas on the publication “Molecular and pharmacological aspects of piperine as a potential molecule for disease prevention and management: evidence from clinical trials [1]”. Tripathi et al. concluded that “*Piperine treatment has also been evidenced to decrease lipid …….* [1].” We agree that piperine might be useful in management of disease. We must carefully assess the conclusions of the current paper by Tripathi et al. because it is a significant report that may be generalized for therapeutic use. The purpose of this correspondence is to provide some additional information that should be examined if piperine is to be utilized in a therapeutic context.

As Ahemed et al. noted, there are few studies on piperine and data on human subjects are limited. A recent report by et al. also showed that piperine is possible useful for management of coronavirus disease 2019 (COVID-19) [2]. A topic that might be a concern that it is not used in human is its toxic property. Piperine can cause liver problem and it can increase toxic effect if administered with other drugs [3, 4]. There is a need for finding for a good pharmaceutic process to prepare a safe piperine before it can be clinically used [3]. This requires a good evaluation on both pharmabiological actions and toxicological profiles of the product derived from the newly proposed pharmacological process.

## Data Availability

Not applicable.

## Abbreviation

COVID-19L: Coronavirus disease 2010
